# A Treatise on the Ligation of the Great Arteries in Continuity

**Published:** 1892-12

**Authors:** 


### Reviews of Books.

*A Treatise on the Ligation of the Great Arteries in Continuity.* By CHARLES A. BALLANCE, F.R.C.S., and WALTER EDMUNDS, F.R.C.S. Pp. xxviii., 568. London: MACMILLAN and Co. 1891.

Considering the number of men at work, and the abundance of opportunities provided for experiment and observation, it is remarkable how few works of the sort before us have been issued from the English press in the last quarter of a century.

Since the time of the Hunters, men like Goodsir, Travers, Abernethy, Guthrie, Bell, and a few others, some of them living, have kept the lamp of scientific surgery alight in England. But for one man, the light in recent years would have indeed been dim: to Lister, and to him almost alone, are we indebted in our own time for upholding the traditions of British surgery, and making our surgical science a guide to all the world.

The work to-day is different from what it was half a century ago. We go deeper, our sight reaches further, our instruments are more precise and more delicate; and as a result more facts have to be observed, and more details of these facts have to be noted, so that in any little department of knowledge the truths to be evolved seem to be almost endless. And in all our English surgical work the spirit of the investigator has not stopped at theoretical knowledge, but has sought to graft practice on knowledge.

The work before us is on the lines and in the spirit of modern surgical research at its best. It collates its material from the widest field possible; it brings to bear on this material all available instruments ; it combines with keen observation a judicious devising of experiments ; and it fittingly concludes with the practical application of the lessons learned to actual work. The work is presented in a manner worthy of its matter. The illustrations, in particular, are most abundant and most beautiful. Having said so much in just praise of the work as a whole, we may be permitted to call attention to some few points in detail.

A presentment of statistics of ligation of the larger arteries shows a mortality which more than justifies any efforts made for its diminution. The objections to experiments on the lower animals are fairly stated and fairly met. The way is further cleared by a chapter on the nature of arteries in gross and minute structure ; and here we meet with a beautiful series of drawings, made according to scale, illustrating the minute structure of all the important arteries of the body. Then follows a series of careful observations of the effect of distension on the arterial walls, and the ground is prepared for a consideration of the process of physiological occlusion, which surgical occlusion, it is inferred, may most properly imitate.

The changes which take place in the physiological closure of the ductus arteriosus, the hypogastric artery, and the umbilical vein are fully described; and Nature's methods of occlusion are further described by reference to the results of arteritis in all its varieties.

It is inferred, indeed it is positively affirmed, that Nature's methods of occlusion, physiological as well as pathological, are the best. Here are the words (p. 78) :—" In conclusion, it may be pointed out to the followers of J. F. D. Jones, that Nature does not think it necessary when occluding the ductus to rupture the two inner coats in order to produce sufficient internal growth; and to the followers of Celsus and Abernethy, that she does not divide the artery to reduce the longitudinal tension—on the contrary, she does not hesitate greatly to increase it; nevertheless, in her hands, failure to occlude very rarely occurs, hemorrhage never."

Between Madam Nature on the one hand, and Messrs. Ballance and Edmunds on the other, Messrs. Jones, Celsus, and Abernethy would seem to cut a sorry figure. Possibly they did their best, according to their lights. As a further conclusion from the pathological study of obliterative arteritis, we are told that "if surgeons would only follow Nature's method they might attain a like success."

There are fallacies here. We deny at the outset that surgery is bound to follow Nature's processes in health or disease. We create a new process, grafted on Nature, which we mould and bend to our will, but we do not blindly follow her. Certainly we should like to have our artificial surgical arteritis as free from hemorrhage as the pathological arteritis; but should we like it to be as frequently followed by gangrene? And when gangrene occurs, do we always act as if Nature's mode of amputation were better than ours? And what is there common to the passive collapse of a disused vessel like the hypogastric artery and its closure, and the violent occlusion of an artery at a seat of ligature, with a pulse throbbing behind it? Pathological arteritis that results in gangrene we do not want; and physiological occlusion of a vessel that has no stream sent through it we cannot get. Might there not be a surgical variety of occlusion better than either?

We are now brought to consider an elaborate study of the conduct and fate of the corpuscles concerned in the formation of the tissue that closes the vessel at the seat of occlusion. The method employed was mainly by the well-known chambers of Ziegler inserted into the tissues of rabbits and guinea-pigs. As studies in the cognate pathology of inflammation in cellular tissue, the observations are of great value; but as justifying the conclusion "that the scar-tissue which occludes the artery is formed, not from the leucocytes of the blood, but from the plasma-cells of the arterial wall" (p. 133), they are not conclusive.

Proof is brought nearer in the subsequent chapters when histological studies of the ligature of rabbits' carotids are detailed. The carotids of rabbits were selected "partly because, the arteries being so small, all the cells of the wall and clot could be readily brought in a living state under the influence of osmic acid . . . partly because complete transverse sections can readily be made of these small vessels; and partly because in them the intima consists . . . of a single layer of endothelial cells resting directly on the elastic lamina, and the process can therefore be viewed in its simplest form." It is just this smallness of size and simplicity of structure that make us doubt the full validity of inferences concerning larger arteries of a complex structure. The inferences are possible, perhaps probable, but they are not, as is asserted, certain.

Subsequent chapters on the conduct and fate of the blood-clot are full of interest, and are worthy of all praise. The results of ligature on the coats of the vessels, and the fate of the aneurism after deligation of its artery, are exhaustively considered and elaborately illustrated.

This leads to the practical side of the question, the best surgical method of deligation in continuity. The authors decide in favour of tying in one place without rupture of the coats. As material for tying they favour either tendon or peritoneal ligature or boiled floss silk. The variety of knot is discussed; and a stay knot is recommended consisting of two or more single reefs, and using the single cords combined into one ligature to finish the knot. Much is made of the slipping of the first hitch in ordinary surgical work, and subsequent failure of the deligation. The criticism is just; but the common want of digital dexterity amongst surgeons, may be met as fitly by superior education of the fingers as by cumbrous additions to the methods of work. We do not know what Mr. Lawson Tait may have to say to the suggested additions to his Staffordshire knot; personally we doubt if it would be possible, with so much increase of friction as must result from the new twistings, to pull the ligature tight enough. The best education would be to see Mr. Tait or any other dexterous surgeon tie the knot a few times.

A series of observations and experiments in actual ligation, with positive instructions as to the conduct of the operation and the management of the patient, in actual cases of ligature, concludes the work.

We believe that the practical conclusions of the authors are sound, and that they ought to guide the practice of the present day. We heartily admit that the original work done and described is of supreme and permanent value; but we consider that on some points conclusions are drawn which are not warranted by the data provided, and on others affirmations are made which are almost certainly wrong. It is easy enough in a general way to say this of almost any great work; as easy to say as it is impossible, short of writing another book, to prove. But, fortunately, it is also easy, and indeed a positive pleasure, to speak of the work as a whole as one which is a credit to British surgery, and which will certainly be a stepping-stone to all future knowledge on the subject with which it deals.

